# Preparation and *In Vitro* Characterization of Triamcinolone Acetonide-Loaded Lipid Liquid Crystal Nanocarriers for Ocular Delivery

**DOI:** 10.34172/apb.43671

**Published:** 2024-12-05

**Authors:** Fatemeh Asgharian Rezae, Malihe Karimi, Hossein Kamali, Bizhan Malaekeh-Nikouei

**Affiliations:** ^1^Student Research Committee, School of Pharmacy, Mashhad University of Medical Sciences, Mashhad, Iran.; ^2^Targeted Drug Delivery Research Center, Pharmaceutical Technology Institute, Mashhad University of Medical Sciences, Mashhad, Iran.; ^3^Department of Pharmaceutics, School of Pharmacy, Mashhad University of Medical Sciences, Mashhad, Iran.; ^4^Nanotechnology Research Center, Pharmaceutical Technology Institute, Mashhad University of Medical Sciences, Mashhad, Iran.

**Keywords:** Triamcinolone acetonide, Lipid liquid crystals, Nanocarriers, Sustained-release formulations

## Abstract

**Purpose:**

This study aimed to develop sustained-release triamcinolone acetonide (TA) formulations using lipid liquid crystals (LLCs) for ocular drug delivery and to characterize the designed formulations.

**Methods:**

Eighteen dispersed LLC formulations were prepared through a top-down approach, incorporating varying concentrations of TA and different proportions of glyceryl monooleate, deionized water, and pluronic F127. An additional formulation comprising TA: hydroxypropyl beta-cyclodextrin (HPβCD) complex was also developed to investigate the influence of HPβCD on the properties of the formulations. The formulations were evaluated for their rheological properties in room temperature using a rheometer, syringeability by passing them through a 27G needle, size measurements via dynamic light scattering (DLS), and morphology through polarized light microscopy (PLM). Furthermore, the prepared formulations were injected into a dialysis tube and placed in a phosphate buffer at pH 7.4 and 37 °C for *in vitro* release evaluation. Samples were taken at predetermined intervals and stored in a refrigerator until HPLC analysis. The percentage of Encapsulation efficacy and drug loading were evaluated using an indirect method. A reversed-phase HPLC method was employed to quantify the drug concentrations in the samples.

**Results:**

All selected formulations demonstrated acceptable parameters, including particle size (less than 200 nm), polydispersity index (PDI) ranging from 0.202 to 0.355, and zeta potential values between -14.3 and -32.8 mV. Additionally, the formulations showed good syringeability and achieved 100% drug release within 48 hours (except for the formulation containing HPβCD). PLM analysis revealed the presence of hexosomes and cubosomes, indicating that an increase in hexosomes contributed to a more uniform drug release from the formulations.

**Conclusion:**

Overall, the study findings suggest that liquid crystalline carriers can be a promising formulation for sustained ocular drug delivery of TA.

## Introduction

 Diseases affecting the posterior eye segment are the leading cause of blindness and vision impairment worldwide. Current treatments for these conditions include anti-angiogenesis agents and corticosteroids, such as triamcinolone acetonide (TA).^1^ However, these prescribed medications face obstacles in effectively reaching the posterior segment of the eye. Topically administered therapies deliver only a minimal quantity to the posterior area, while systemic medications must traverse the blood-retina barrier. This passage often requires higher dosages, leading to increased side effects.^2,3^ Intravitreal injection (IVI) is a highly effective method for delivering medication to the posterior segment of the eye. However, many patients find it undesirable due to its invasive nature, high costs, and potential complications associated with repeated injections. These side effects highlight the need for sustained-release formulations that can minimize the frequency of injections. Increasing the bioavailability of topical drugs can also pave the path to treating posterior eye segment disease using this comfortable and non-invasive method.^4^

 TA is a relatively safe and effective conventional corticosteroid for treating ocular diseases that require long-term steroid therapy, such as diabetic cystoid macular edema, macular edema secondary to retinal vein occlusion, pseudophakic macular edema, uveitis, proliferative vitreoretinopathy, and exudative age-related macular degeneration.^5^ However, delivering effective doses of TA to the posterior segment of the eye remains challenging. The conventional form of TA injected intravitreally is a 40 mg/mL suspension with the FDA-approved, preservative-free formulation, Triesence^TM^, currently in the market.^6^ Nevertheless, this form has complications of IVI such as increased intraocular pressure, hemorrhage, endophthalmites, and cataract.^7^ Efforts have been made to design novel topical formulations of TA to increase bioavailability of topical formations and alleviate the need for intraocular injections. Thus far, studies have been conducted on liposomes,^8^ emulsomes,^9^ nanocapsules,^10^ PLGA-chitosan,^11^ and poly-beta amino ester nanoparticles containing TA.^12^ Furthermore, Topical Lipid-based nanoparticles have enhanced bioavailability due to higher residence time on the ocular surface and enhanced corneal penetration.^13^

 LLC are lipid-based carriers that form by self-assembling amphiphilic molecules (i.e., certain lipids) in an aqueous environment. LLCs, based on solvent content (lyotropic) or temperature (thermotropic), appear in various phases and are capable of phase transition. Dispersed LLC are nanoparticles formed by high-energy dispersion of hydrated lipids in an aqueous solution (top to down) or controlled addition of a lipid solution to aqueous phase (down to top). Phases in dispersed LLC include lamellar, hexosomes, and cubosomes, which appear accordingly depending on the increase of solvent content, and reverse phases can also form by a change in the solvent’s polarity.^14^ The amphiphilic nature of LLC facilitates the loading of various drugs with a wide range of polarity. At the same time, the unique structures of lipidic regions and aqueous channels allow for the sustained release of loaded drugs. Furthermore, their lipidic nature enhances the penetration through various mucosa and cell membranes, resulting in a higher drug bioavailability.^15^ Lipids used in LLCs are generally biocompatible, biodegradable, and readily available at a low cost.^16^ These characteristics capacitate LLCs as valuable candidates for ocular drug delivery, either in the form of intraocular injection or topical application.

 LLC-based formulations have shown superior performance in various drug formulations. Chen et al. developed a topical formulation of cyclosporin, that not only demonstrated no eye irritation or corneal damage but also indicated the potential of LLCs in reducing drug toxicity.^14^ A cubosome formulation of flurbiprofen was found to have lower eye irritation and increased bioavailability.^17^ Ketorolac-loaded cubosomes showed twice the corneal penetration compared to ketorolac solution and released the drug within 20 hours, while the plain solution fully released the drug in 2 hours.^18^ Furthermore, Timolol maleate cubosomes had higher corneal penetration and retention time than the commercially available eye drops and lowered the intraocular pressure more effectively.^19^

 LLC formulations have shown promise in sustained drug delivery and increasing the bioavailability of various drugs; however, dispersed LLC formulations of TA have yet to be investigated. In this study, we aimed to design and develop dispersed LLC formulations of TA and to evaluate them using various in-vitro methods.

## Materials and Methods

###  Materials

 TA was purchased from Exir Pharmaceuticals, Iran. Glyceryl monooleate (GMO) and phosphatidylcholine (PC) were all purchased from Tinab Shimi, Iran. HPLC grade solvents, including methanol, acetonitrile, and ethanol (96%), were purchased from second-hand providers, initially supplied by Deajung Chemicals & metals Co, Korea. Hydroxypropyl-β-cyclodextrin (HPβCD) and pluronic F127 were obtained from Shanghai Honglian Chemical Technology, China, and Merck, Germany, respectively.

###  Preparation of dispersed LLC formulations

 According to the composition detailed in Table 1. The dispersed LLC formulations of TA were prepared using an up-to-down method.

**Table 1:** Composition of dispersed LLC formulations containing TA

**Formulation**	**TA concentration**	**Content (w/w%)**			
		**GMO**	**PO sol**	**DW**	**Other content**
F1	0.4% w/w	3.5	95	1.5	
F2	0.4% w/w		99.6		
F3	0.05% w/w	3.5	95	1.5	
F4*	0.05% w/w	3.5	95	1.5	
F5*	0.07% w/w	3.5	95	1.5	
F6*	0.1% w/w	3.5	95	1.5	
F7*	0.2% w/w	3.5	95	1.5	
F8*	0.3% w/w	3.5	95	1.5	
F9*	0.1% w/w	3.5	95	1.5	
F10	0.3% w/w	3.5	95	1.5	
F11	0.03% w/w	3.5	95	1.5	
F12	0.05% w/w	4.9	93	2.1	
F13	0.05% w/w	6.3	91	2.7	
F14	0.05% w/w	2.5	95	1.5	1% PC
F15	0.05% w/w	1.5	95	1.5	2%PC
F16	0.05% w/w	4	93	2	1%PC
F17	0.05% w/w	3.47	94.4	1.49	0.61% HPβCD
F18	0.05% w/w	3.49	94.76	1.49	0.25% HPβCD

Abbreviations: TA, Triamcinolone acetonide, GMO, glycerin monooleate, PO, pluronic F127 GEL, lipid liquid crystal gel, HPβCD, Hydroxypropyl-β-cyclodextrin, DW, deionized water Formulations marked by * were prepared using a different method as described.

**Figure 1:**
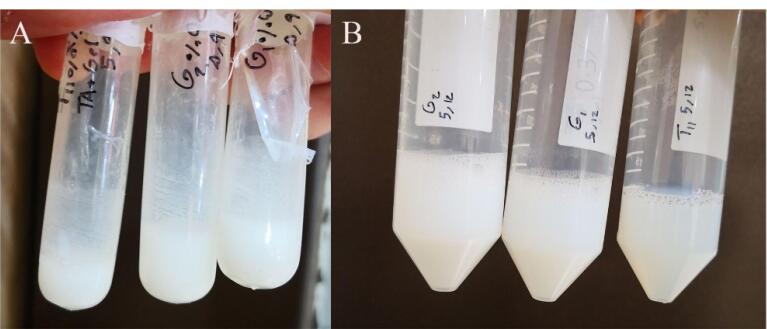
Appearance of the dispersed LLC formulations. (A) First step: TA, GMO, and DW were weighed and mixed, (B) Second step: The LLC gel prepared in the previous step was mixed with F127 solution

 First step: TA, GMO, and DW were weighed and initially mixed manually with a micro spatula. Subsequently, the mixture was further homogenized using a microtube shaker at 20 000 rpm for 5 minutes. The resulting formula was then subjected to bath sonication at 50 °C for 30 minutes (Ultrasonic cleaner 2200 ETH S3, Soltec, Italy). In order to prevent lipid oxidation, the microtube containing the formulation was filled with nitrogen and sealed before any processing steps. The prepared formulations were then stabilized at room temperature (25 °C) for one week (Figure 1). Additionally, three formulations (F14-16) were created by incorporating PC into the lipid phase to assess the impact of PC on the formulations.

 Another method of preparation was also investigated. In this method, TA and GMO were initially physically mixed and bath-sonicated at 50 °C for 30 minutes. After sonication, DW was added to the mixture without any additional mixing. The resulting formulation was then equilibrated at room temperature for a duration of one week. Subsequently, the impact of this method on the physicochemical properties of the formulations was analyzed, and the results were used to determine the optimal preparation method. The asterisk (*) in Table 1 indicates the formulation prepared using this method.

 Second step: Pluronic solution was prepared by adding 10 g of F127 to 100 g of DW and keeping it in the refrigerator (8 °C) for 20 minutes. Pluronic F127 can be solved in water either by heat or refrigerating as its solubility increases by either change in temperature.^20^ The LLC gel prepared in the previous step was heated to 60 °C and mixed with F127 solution. Next, the formula was homogenized at 2000 rpm for 10 minutes (Ultra-turbax IKA T110, IKA, China) and then probe sonicated using Soniprep 150 (MSE scientific, UK) at maximum power for 5 minutes (Table 1, Figure 1).

 Two formulations (F17, F19) were prepared by adding HPβCD to evaluate the effect of HPβCD on drug release from the formulations. The choice of the cyclodextrin type was based on previous research.^21^ First, TA was fully solubilized in 70% ethanol, while HPβCD was dissolved in distilled water. These two solutions were mixed and left at room temperature for three days to allow for the solvents’ evaporation. Simultaneously, a hydrated lipid mixture was prepared by mixing GMO and DW, followed by bath sonication at 50 °C for 30 minutes. A week later, the F127 solution was added to the dried TA: HPβCD mixture and bath sonicated at 30 °C for 10 minutes to dissolve the drug: CD complex in the aqueous phase. The drug-free lipid liquid gel that was previously prepared was heated to 60 °C and then mixed with the aqueous phase containing the TA: HPβCD complex. Finally, the formulation was homogenized using the previously described method in the beginning of this section.

###  HPLC method for TA analysis

 A stock solution of TA was prepared by solving TA in methanol (1 mg/mL) and later diluted to a series of concentrations ranging from 0.048 to 50 µg/mL. These solutions were analyzed using the Shimadzu LC-20AD HPLC system (Shimadzu, Japan), connected to a UV/VIS spectrophotometer (SPD-20AD) set at 254 nm. A Knauer reverse phase C18 4.6 mm ID, Nucleosil-100, 150 mm column (Knauer, Germany) with a mobile phase of 40% acetonitrile, 60% deionized water, at a flow rate of 0.8 ml/min was used for chromatographic separation.^22^ The injection volume was 20 µl with column temperature set at 25 °C. Subsequently, the equation for the calibration curve was obtained by plotting the absorbance peak areas of TA against the corresponding drug concentrations. The limit of detection (LOD) and limit of quantification (LOQ) were also calculated using the below formulas^23^:


$$LOD=\frac{3.3\times \sigma }{S}$$

$$LOQ=\frac{10\times \sigma }{S}$$

Where σ is the Standard deviation, and *S *is the slope of the drug’s standard curve. The data below LOQ were removed from the calculations.

###  Physiochemical characterization of dispersed LLC formulations

 Dispersed formulations were selected for further assessment based on the amount of drug sediment observed and the overall appearance of each formulation.^24^ Formulations that exhibited minimal drug sediment, highest possible drug loading, and a homogenous white appearance were chosen for additional assessment.

####  Determination of particle size, size distribution, and zeta potential

 The mean size, polydispersity index (PDI), and zeta potential of seven selected formulations were evaluated using dynamic light scattering (DLS) (Zetasizer nano-zs, Malvern Instruments, UK) at 25°C, with measurements conducted in triplicate. These seven samples were chosen to investigate how changes in various parameters affect the formulation. These parameters included alterations in drug concentration (F3, F4, F10), increasing GMO content (F13), addition of PC (F14, F16), and CD (F18).

####  Measurement of encapsulation efficacy and drug loading

 The percentage of Encapsulation efficacy and drug loading were evaluated using an indirect method.^24^ The selected formulations were centrifuged at 1500 rpm for 10 minutes (MIKRO 120 Hettich zentrifugen, ohne Rotor, Germany) to separate the unloaded drug.^25^ Afterward, the formulations (supernatant liquid) and sediment were dissolved and diluted in methanol.^26^ The diluted solutions were then analyzed for TA content using the previously described HPLC method by Wallace et al.^27^ The encapsulation efficiency (EE%) and drug loading were calculated using the following formulas:


$$Encapsulation=\frac{D_{total}-D_{unloaded}}{D_{total}}\times 100$$

$$Loading=\frac{D_{total}-D_{unloaded}}{F_{total}}\times 100$$

Where D_total_ is the initial amount of TA in formulation, D_unloaded_ is the precipitated drug formulation, and F_total_ is the total weight of the formulation.

####  Viscosity measurements and syringeability

 The rheological behavior of the selected formulations was investigated using a Brookfield R/S plus (Brookfield, UK) in Rot stairs mode at 25 °C, using a 3 ml steel cone. The formulations were exposed to 11 steps of continuous shear stress ranging from 0 to 100 Pa. Viscosity was measured using the power law set by the program. All measurements were carried out in triplicate. Moreover, all formulations were passed through a 27G needle syringe to assess their syringeability.

###  Polarized light microscopy (PLM)

 A microscope slide of a single drop of the selected formulations was prepared and subsequently analyzed using a polarized light microscope (Olympus BH2, Olympus Life Sciences, Japan) connected to a digital camera. This setup was used to evaluate the morphology of the prepared formulations.

###  Measurement of TA solubility 

 Drug solubility is essential for determining the appropriate volume of the release study environment; therefore, an excess amount of TA was dissolved in PBS at pH 7.4 and maintained at 37 °C. The combination of TA: HPβCD in PBS was also evaluated to assess the effect of CDs on TA solubility. Samples were then centrifuged to separate any undissolved drug, and the supernatant was measured for drug content using the HPLC method. All samples were prepared and evaluated in triplicate.

###  In vitro release study of dispersed LLC formulations

 The selected formulations were vortexed at 20 000 rpm for 5 minutes prior to injection to ensure no sediment remained in the formulation. A 12KD dialysis tube was cut into 5 cm pieces and tied on one end. Then, 3 mL of the dispersed formulation was then injected into the tube and sealed on the other end. The tube was placed in 120 mL of PBS (pH 7.4) at 37 °C and shaken in a shaking water bath (Shimaz, Iran) at 40 rpm. Approximately 0.5 ml of the release environment was sampled at predetermined time points (1, 2, 4, 6, 8, 12, 24, 36, and 48 hours) and replaced with an equal volume of PBS to maintain the sink condition. The samples were then refrigerated until they were measured for TA content using HPLC.

###  Statistical analysis 

 The results were analyzed using GraphPad Prism 10, and related graphs were also drawn as needed. To compare release similarity between formulations, the similarity factor (f2) was calculated based on previous research.^28^ An ordinary one-way ANOVA with Tukey’s multiple comparison test was utilized as required, and p values less than 0.05 were considered statistically significant.


$$f_{2}=50\log\left\{{\left[1+\frac{1}{n}\sum_{t=1}^{n}{\left(R_{t}-T_{t}\right)}^{2}\right]}^{-0.5}\times 100\right\}$$

## Results

###  Calibration curve of TA

**Figure 2:**
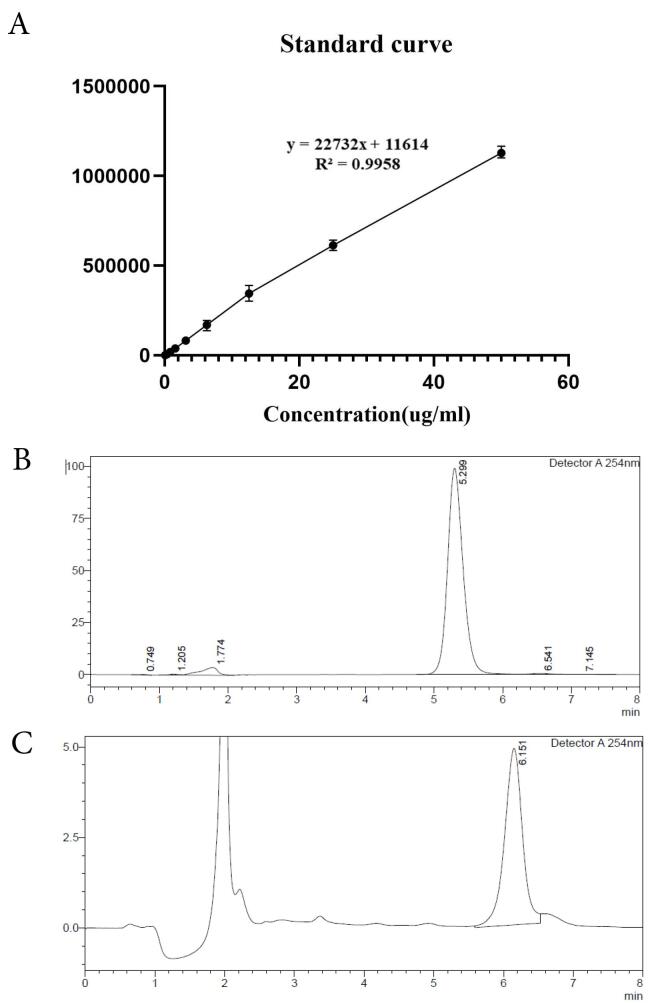
(A)HPLC standard curve and chromatograms of TA. (B) HPLC chromatogram of TA (50 µg/mL) in methanol, (C) HPLC chromatogram of TA in PBS (F13 release sample at 6 h)

 The calibration curve of TA showed linearity in the range of 0.39 to 50 µg/mL with a regression value of 0.995 (Figure 2A). The chromatogram of 50 µg/mL concentration of TA in methanol is shown in Figure 2B, with a retention time of 5.29 minutes in methanol and 6.15 in PBS (pH 7.4). Furthermore, an example of release sample analysis is presented in Figure 2C. The peaks observed in both chromatograms are sharp and with an acceptable retention time. Moreover, the other peaks observed in Figure 2 may represent buffer, NMP, and lipid peaks as this is a release sample analysis and contains all mentioned substances. The calculated LOD and LOQ were 0.132 µg/mL and 0.402 µg/mL, accordingly.

###  Physiochemical characterization of dispersed LLC formulations

 Four dispersed formulations (F3, F13, F14, F18) were selected for further evaluation based on the amount of drug sediment and the overall appearance of the formulation.^24^ These selected formulations had the maximum possible drug loading with the minimum amount of drug sediment and a homogenous white appearance as shown in Figure 1.

####  Dynamic light scattering

**Figure 3:**
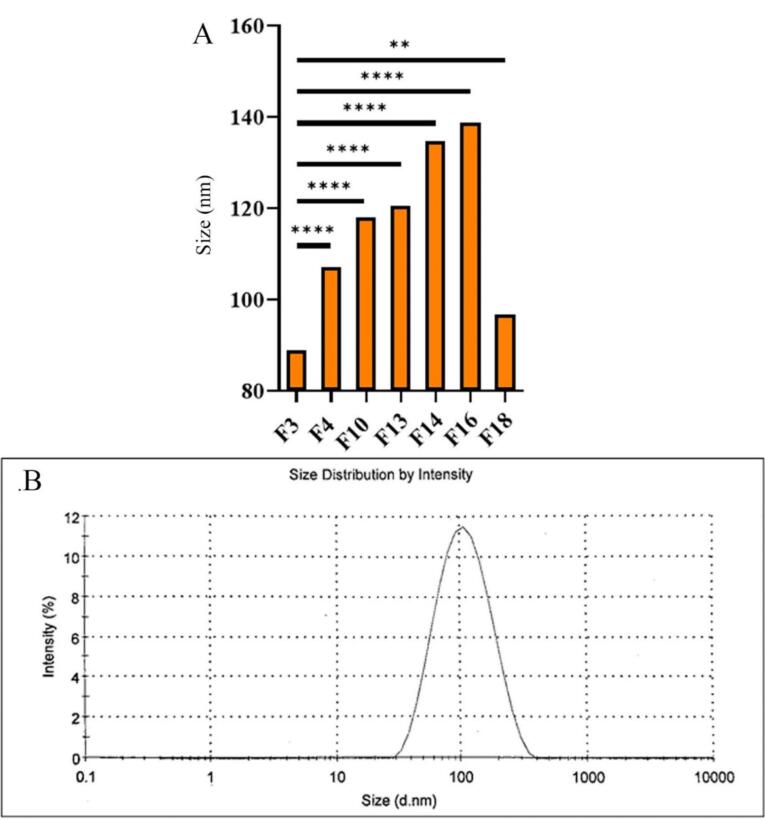
(A) Particle size comparison of dispersed formulations, (B) Size distribution diagram of F3 dispersed LLC formulation

 Seven formulations were chosen to investigate the effects of various parameters on their characteristics. These parameters included changes made in drug concentration (F3, F4, F10), increasing GMO content (F13), addition of PC (F14, F16), and CD (F18). The DLS results of these formulations, mean size measurements, zeta potential, and PDI, are shown in Table 2. A comparison of the particle size between various formulations is also shown in Figure 3A. Furthermore, an example of a sample PDI diagram is presented in Figure 3B.

**Table 2:** Particle size, size distribution, and zeta potential of dispersed LLC formulations

**Formulation**	**Z. Average (nm)**	**PDI**	**Zeta potential (mV)**
F3 (0.05%)	89.01 ± 0.21	0.217 ± 0.021	-20.7 ± 0.4
F4 (0.05%)	107.04 ± 0.14	0.256 ± 0.009	-25.5 ± 0.1
F10 (0.3%)	118.10 ± 0.09	0.312 ± 0.015	-38.5 ± 0.8
F13 (0.05%)	134.82 ± 0.20	0.238 ± 0.030	-16.4 ± 0.3
F14 (0.05%)	138.73 ± 0.14	0.355 ± 0.102	-32.8 ± 0.2
F16 (0.05%)	141.10 ± 0.31	0.356 ± 0.081	-28.4 ± 0.3
F18 (0.05%)	96.70 ± 0.18	0.202 ± 0.041	-14.3 ± 0.5

 The particle sizes of the designed formulations were found to vary between 89.01 ± 0.21 and 141.10 ± 0.31 nm, which are all within the acceptable nanoparticle range,^29^ with a PDI of 0.202-0.356. Furthermore, increases in drug content (F3, F4, and F10), the addition of CDs (F18), increases in lipid concentration (F13), and the addition of PC (F14, F16) to the formulations considerably increased the mean particle size. However, the addition of CDs had a less considerable impact. The other preparation method (formulations marked with asterisk*) used to produce the formulation also increased particle size and, therefore, was no longer used to develop dispersed formulations in this study.

 Furthermore, it was observed that the PDI increases with higher drug concentration (F10), an increase in lipid content (F3), and the addition of PC to the formulation (F14, 16).

####  Encapsulation and loading measurements

 The Encapsulation Efficacy and drug loading of the selected formulations were measured, and the sediment in each formulation represents the unloaded drug (Table 3).^25^

**Table 3:** Loading and Encapsulation efficacy of dispersed formulations

**Formulation**	**Drug content (mg)**	**Loading (%)**	**EE (%)**
F3	1.85	0.0185 ± 0.031	37.05 ± 0.05
F3 sediment	1.20	**-**	**-**
F13	1.50	0.0150 ± 0.052	30.10 ± 0.12
F13 sediment	3.63	**-**	**-**
F14	1.36	0.0136 ± 0.048	27.22 ± 0.08
F14 sediment	1.03	**-**	**-**
F18	2.10	0.0210 ± 0.104	42.31 ± 0.14
F18 sediment	1.43	**-**	**-**

 The objective of designing nanocarriers is to reach the maximum possible loading and encapsulation efficacy. According to the loading and EE% measurements, F3 and F18 (containing HPβCD) demonstrated the best results in comparison, and can be considered optimal. It was noted that increasing the content of GMO (F13) and adding PC (F14) led to a reduction in both drug loading and EE%. However, the centrifugation process used to separate the unloaded drug may have affected the results.

####  Viscosity measurements and syringeability of dispersed LLC formulations

**Figure 4:**
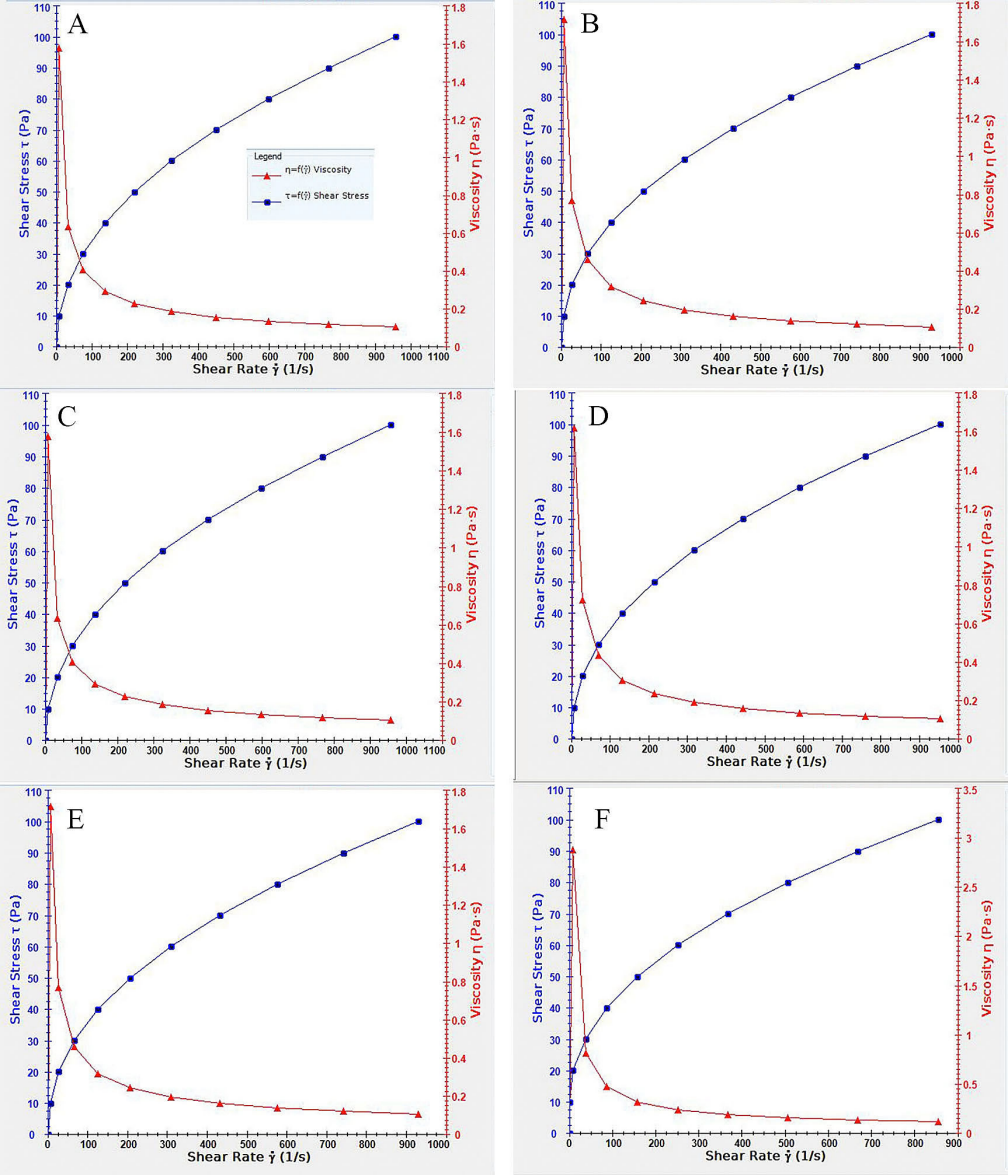
Viscosity diagramof selected dispersed LLC formulations. (A) F14, (B) F3, (C) F13, (D) F18, (E) F3 in 34 degrees °C, (F) F3 in room temperature (25°C)

 The viscosity diagrams of all selected formulations at 25 °C, and the viscosity diagram of F3 at corneal temperature (34 °C)^30^ are shown in Figure 4.

 All formulations exhibited pseudoplastic behavior, where the viscosity decreases with increased shear stress in a non-linear pattern.^31^ This feature can be favorable in designing topical formulations in the form of eye drops, as the viscosity decreases when applying the eye drop by increasing users’ pressure on the container. When applied, the lack of external pressure results in higher viscosity and helps retain the formulation in the applied location.^32^ An increase in viscosity was also observed by increasing temperature, which can be helpful as the higher viscosity stabilizes the formulation in place and prevents the dispersion of the medicine when applied topically. Furthermore, the addition of PC also increases the viscosity, which can be due to the physiochemical characteristics of PC and its intrinsic high viscosity and melting point.

###  Triamcinolone acetonide solubility 

 The solubility of TA and TA: HPβCD complex in PBS was measured at 24.66 ± 0.25 and 153.74 ± 0.32 µg/mL, respectively. The solubility results were used to calculate the suitable volume of the release medium needed to maintain the synchronization conditions, which should be capable of solubilizing 2-10 times the whole drug content in the release medium.^33^ As anticipated, the addition of CDs considerably increased the drug solubility of TA, which is only sparingly soluble in aqueous buffers. It is well established that CDs are commonly employed to increase the solubility of such drugs in aqueous environments.^21^

###  PLM photography

**Figure 5:**
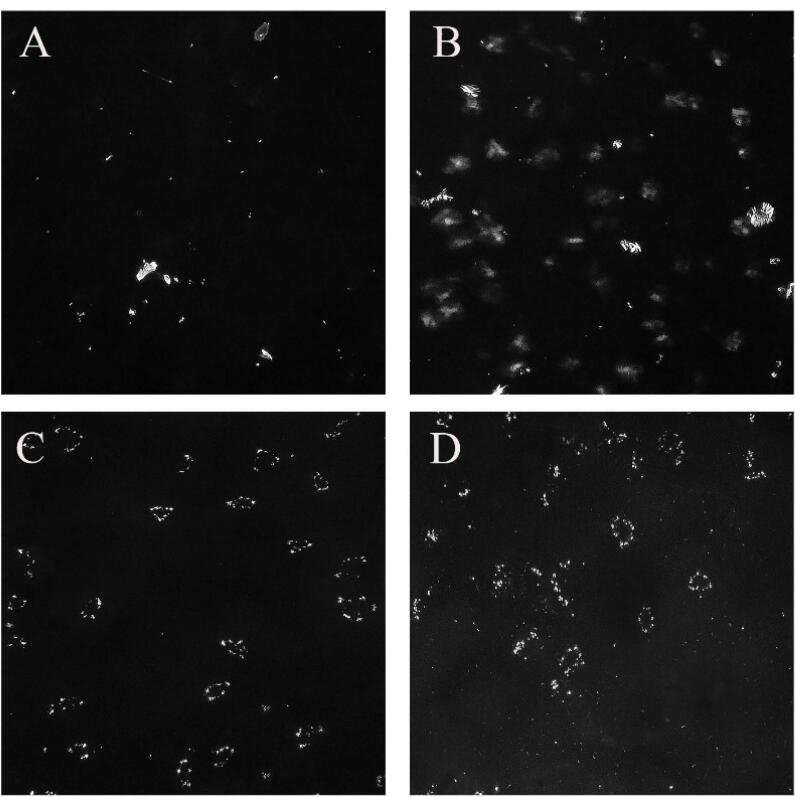
PLM photography of dispersed formulations (×1000). (A) F3, (B) F18, (C) F13, (D) F14. Arrows show examples of anisotropic forms of LLC (hexosomes, lamellar), the black background is formed of isotropic LLC (cubosomes)

 The images of dispersed LLC formulations captured using PLM are displayed in Figure 5. It has been observed that LLC appear in 2 forms in PLM photography: isotropic (i.e., Cubosome) which allows all light to pass through and appears entirely black, while anisotropic (i.e., hexosome, lamellar) which reflect light and appears as bright crystals.^34^ As shown in Figure 5, the F13 formulation exhibits the highest number of bright crystals (anisotropic forms), while F3 is predominantly in the isotropic state (cubosomes). Additionally, the phases present in dispersed LLC significantly influence the formulations’ release, loading, and overall characteristics.

###  In-vitro release of dispersed formulations

**Figure 6:**
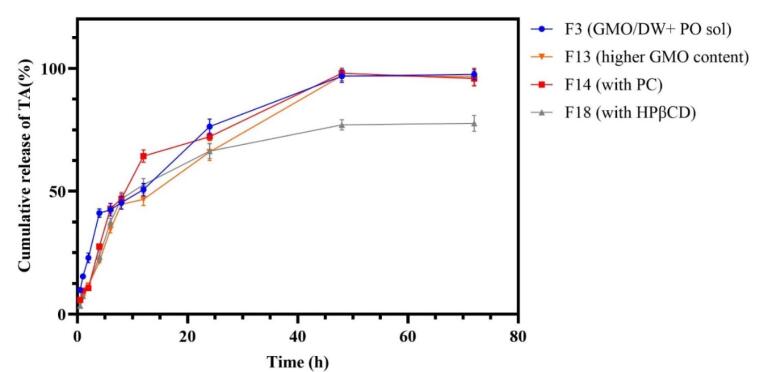
TA release from dispersed LLC formulations. F3 (3.5% w/w GMO), F13 (6.3% w/w GMO), F14 (2.5% w/w GMO, 1% w/w PC), F18 (with 0.25% w/w HPβCD)

 The drug release results from dispersed formulations are represented in Figure 6. All formulations, except for F18, released their entire drug content within 48 hours. In contrast, F18 did not fully release its drug content even after 72 hours. It was observed that F13 showed slower and more consistent drug release, while formulations F3 and F14 displayed a burst release and sudden changes in their release profiles, respectively. In order to facilitate comparison of drug release among various formulations, the similarity factor (f2) was calculated using the formula described in the methods section (Table 4). Research has shown that the similarity factor indicates the likeness of drug release from different formulations, with values above 50 suggesting a similarity in release profiles at a specific time. As illustrated in Table 4, F18 (containing HPβCD) has demonstrated a significant difference in release compared to all other formulations over the past 48 hours. Specifically, it differs from formulation F3 at 2 and 4 hours. In order to investigate the burst release from various formulations, data from the first 6 hours were analyzed separately for f2 using Ordinary One-way ANOVA and Tukey’s multiple comparison test. The findings are presented in Table 5. To effectively understand the results of the similarity factor, one side is designated as the base, while the others are compared against it. For instance, in the comparison of f2 (F3-F18) with f2 (F18-F13), F18 serves as the base. In this case, we compare formulations F3 and F13.The results indicate that F13, which has a higher GMO content, and F18, which contains HPβCD, exhibit lower burst release rates compared to F3, with a *P* value of less than 0.05.

**Table 4:** Similarity factor (f2) for TA release comparison

**Time (h)**	**f2 (F18-F13)**	**f2 (F14-F18)**	**f2 (F14-F18)**	**f2 (F3-F13)**	**f2 (F3-F18)**	**f2 (F3-F14)**
0.5	95.57	85.92	79.72	65.74	62.83	74.30
1	91.36	73.25	81.04	52.14	54.94	61.77
2	99.31	89.86	92.60	47.33	46.85	45.07
4	78.41	60.26	71.14	35.39	38.37	43.56
6	69.55	52.81	65.52	54.94	69.43	94.33
8	73.74	75.50	99.21	85.34	87.12	89.96
12	73.30	40.29	45.38	88.63	83.31	42.27
24	99.99	80.97	80.85	56.61	56.56	64.11
48	33.58	99.37	33.83	97.25	33.04	94.83
72	33.89	94.93	34.71	99.94	33.97	95.65

**Table 5:** Comparison of Similarity factor (f2) in the first 6 hours of TA release using ANOVA

**Tukey's multiple comparisons test**	**Mean Diff.**	**95.00% CI of diff.**	**Adjusted P value**	**Summary**
f2(F3-F14) vs. f2(F3-F18)	9.326	-18.84 to 37.49	0.9053	ns
f2(F3-F14) vs. f2(F3-F13)	12.70	-15.47 to 40.87	0.7301	ns
f2(F3-F14) vs. f2(F14-F18)	-14.20	-42.36 to 13.97	0.6321	ns
f2(F3-F14) vs. f2(F14-F13)	-8.611	-36.78 to 19.56	0.9304	ns
f2(F3-F18) vs. f2(F3-F13)	3.372	-24.79 to 31.54	0.9990	ns
f2(F3-F18) vs. f2(F14-F18)	-23.52	-51.69 to 4.646	0.1406	ns
f2(F3-F18) vs. f2(F18-F13)	-32.36	-60.53 to -4.192	0.0179	*
f2(F3-F13) vs. f2(F14-F13)	-21.31	-49.48 to 6.858	0.2177	ns
f2(F3-F13) vs. f2(F18-F13)	-35.73	-63.90 to -7.565	0.0075	**
f2(F14-F18) vs. f2(F14-F13)	5.584	-22.58 to 33.75	0.9890	ns
f2(F14-F18) vs. f2(F18-F13)	-8.83	-37.01 to 19.33	0.9229	ns

ns: non significant, *P*>0.05;*: *P*≤0.05; **: *P*≤0.01.

###  Optimum dispersed formulation

 Based on the results from the conducted in vitro examinations, the F13 formulation was selected as the optimal formulation. This decision was made because it demonstrated complete drug release within two days, along with a slow and steady release profile attributed to the highest formation of hexagonal LLC. Conversely, F18 exhibited incomplete drug release, likely due to the formation of the drug CD sediment, and therefore was not chosen. Additionally, F14 displayed a more irregular drug release pattern, and F3 had a higher burst release compared to F13 formulation.

## Discussion

 Diseases of the posterior eye segment are among the leading causes of eye impairment and blindness worldwide. Treatment for these conditions primarily relies on corticosteroids and antiangiogenic agents.^35^ However, the topical application of these medications exhibits minimal absorption in the posterior parts of the eye. Additionally, the presence of the blood-retinal barrier poses a significant challenge to the absorption of drugs administered systemically. While intraocular injections are highly effective, this method is invasive, costly, and complex, which can lead to decreased patient compliance.^2,3,36^ Therefore, it seems necessary to design and manufacture sustained-release formulations. Moreover, designing topical formulations with higher bioavailability can reduce the need for invasive methods, thereby enhancing patients’ compliance and the ease of medication administration. In this study, we designed dispersed lipid liquid crystal (LLC) formulations of TA that can provide a sustained release of TA over a period of 48 hours.

 Dispersed LLC systems can be suitable for designing sustained-release intraocular drug delivery due to their ease of injection, pseudoplastic rheological behavior, and reduced drug toxicity. Furthermore, the lipids used in these formulations are biodegradable, eliminating the need for surgery to remove any remnants from the eye.^16^ Moreover, the amphiphilic structure of their compounds allows the loading of various drugs regardless of their polarity. Studies have been conducted on dispersed LLCs of several drugs, such as ondansetron,^24^ ketorolac,^18^ cyclosporin,^37^ timolol,^19^ and vancomycin.^38^ However, dispersed formulations typically exhibit a rapid drug release, often reach maximum release within 24 - 72 hours. Therefore, using these formulations in a topical form is a more favorable option.

 Moreover, the dispersed formulation’s lower viscosity and liquid form make it easier to use as eye drops. Although there are differences between in-vivo and in-vitro drug release due to the higher viscosity of the vitreous humor and the unique conditions of the internal environment of the eye,^39^ *in vivo* studies also confirm this release pattern. Therefore, such formulations should be considered primarily as a topical drug delivery option. Furthermore, unlike other diacyl lipids, GMO LLC phases dissolve more quickly in aqueous environments and can exchange monomer lipids with other biological structures. This ability disrupts biological membranes and enhances penetration, improving the application of the GMO-based dispersed LLC in eye drops.^26^

 The lipid GMO can form cubic and hexagonal phases depending on the amount of solvent and temperature. In physiological conditions and excess water, the cubic phase formation is more commonly observed,^40^ which can also be seen in PLM images. Similarly, in dispersed formulations made of GMO, water, and pluronic F127 for the drugs simvastatin, oral indomethacin, and ophthalmic sertaconazole,^32^ the predominance of the cubic phase has been demonsterated.^41^ However, as the lipophilicity of the formulation increases due to a higher concentration of GMO (sample F13), the amount of water in the medium decreases, leading to the formation of a hexagonal phase. These results align with the study conducted by Salonen et al, which found that in self-assembled monoglyceride-based dispersions at room temperature with excess water, an increase in oil content results in the formation of cubic and hexagonal phases.^42^ On the other hand, PC in the LLC structure primarily tends to form a lamellar phase. This tendency can be affected by the presence of other lipids in the formulation.^43^ With the addition of PC to the dispersed formulation (F14 sample), lamellar formations can be observed in the PLM images.

 Among the dispersed LLC, hexosomes release the drug slowly and continuously due to their limited aqueous channels. In contrast, cubosomes, which have a higher solvent content and more extensive aqueous channels, provide a relatively faster drug release.^40^ With an increase in lipid percentage in the F13 sample, resulting in the formation of hexosomes, we observe less burst release and a more uniform drug release process compared to the F3 formulation, which has a lower content of GMO. Similarly, a study involving Vancomycin dispersed LLC found that the release from the hexagonal phase was prolonged in simulated tear fluid, unlike that from the cubic phase. This was attributed to the hexagonal phase’s two-dimensional symmetry and its closed water channels, which create a complex diffusion route that facilitates the gradual release of the trapped molecule.^38^ On the other hand, the PC lamellar phase tends to change depending on the content of water, other lipids, and even the amount of drug in the formulation,^40,43^ and this matter affects the drug release from PC-containing LLC formulations. As seen in sample F14, the release pattern is somewhat irregular, which can be a sign of the phase change of the formulation following the alterations made during the drug release process.

 Cyclodextrins (CDs) feature a hydrophobic inner core and a hydrophilic outer shell, primarily used to enhance the solubility of insoluble drugs in aqueous environments. The application of CDs in ophthalmic formulations has been explored in numerous studies, with HPβCD being utilized in the development of formulations containing dexamethasone, fluorometholone, and fluocinolone.^44^ Additionally, a separate study investigated the enhancement of TA solubility using HPβCD. This study demonstrated that a molar ratio of 1:7 (drug: CD) resulted in a homogeneous molecular structure, whereas a lower drug-to-CD ratio led to the formation of drug crystals measuring 10 to 15 microns.^21^ Due to the impracticality of employing a 1:7 molar ratio of drug to CD (which would require 124 g of CD for a total formulation mass of only 10 g), the reduced quantities of CD used are likely to promote crystal formation alongside the drug. This crystallization may contribute to the lower release rates observed in the F18 formulation. The F18 formulation shows higher loading and encapsulation, but the decreased release further supports this hypothesis.

 Our designed formulations exhibited particle sizes ranging from 89.01 ± 0.21 nm to 141.1 ± 0.31 nm, all of which fall within the acceptable nanoparticle range. The PDI ranged from 0.202 to 0.356. Particle sizes smaller than 200 nm are known to penetrate the cornea effectively, making them ideal for ophthalmic formulations.^45^ In comparison, our particle sizes were significantly smaller than those reported in studies on diclofenac sodium,^46^ latanoprost,^47^ and sertaconazole^32^ cubosomes with particles size ranging between 409 ± 3.0 to 679 ± 2.0 nm, 204.6 ± 1.6 to 217.8 ± 19.6 nm, and 125.10 ± 1.41 to 383.50 ± 7.78 nm, respectively. The preparation method plays a crucial role in determining the particle size of dispersed LLC formulations. Formulations created using a bottom-up approach often yield larger particle sizes.^46^ In our study, we observed that a simple change in production method and removal of a single homogenizing step resulted in larger particle size (F3 vs. F4).

 The PDI increases with higher drug concentrations (F10), an increase in lipid content (F3), and the addition of PC to the formulation (F14, 16). The increased viscosity resulting from the addition of PC could interfere with the homogenization process, leading to larger particle sizes. Younes et al suggested that a higher concentration of lipid phase could cause aggregation of the formed nanoparticles,^32^ which was further confirmed by a study conducted by Malaekeh-Nikouei et al on fluorometholone cubosomes.^48^ Additionally, a higher drug concentration and the inclusion of HPβCD, specifically with TA, could also result in particle aggregation since TA naturally tends to form crystals.^49^ The literature has already established the formation of TA: HPβCD complexes.^21^ Furthermore, a zeta potential greater than 20 mV or less than -20 mV is considered optimal for preventing aggregation.^50^ Almost all our selected dispersed formulations (F3, F13, F18) exhibited zeta potentials within this optimal range.

 Our study was the first to develop a dispersed LLC formulation of TA and to evaluate impact of adding HPβCD on the physiochemical characteristics and drug release from this formulation. We successfully produced a number of formulations and explored how variations in the preparation method, drug concentration, and types of lipids affected the LLC formulations. Unfortunately, performing in-vivo examinations was not feasible in our study. Future research could focus on assessing the safety and efficacy of these formulations in retinal cell lines and rabbit eyes. Furthermore, long-term stability studies were not conducted, which can also be considered in future work.

## Conclusion

 In this study, sustained-release formulations of TA were prepared using a dispersed LLC system for ocular use to overcome the disadvantages of traditional drug forms. The optimum formulation, F13 (6.3% w/w GMO, 91% pluronic F127), with a drug concentration of 0.05% w/w, released its total drug content within 48 hours. The formation of hexosomes was observed in this formulation, which resulted in a more uniform drug release. Considering the unique characteristics of LLCs, these formulations can treat various eye diseases requiring corticosteroid treatment.

 To the best of the authors’ knowledge, this study was a comprehensive pilot research on developing a dispersed LLC formulation of TA. The research also evaluated the impact of adding HPβCD on the physicochemical properties and drug release characteristics of this formulation. A satisfactory number of formulations were produced in this research, and the effects of variations in the preparation method, drug concentration, and lipid composition on the LLC formulations were also investigated. Given the potential of topical lipid-based nanoparticles for their enhanced bioavailability and biocompatibility, this formulation should be considered for further research in ocular drug delivery. However, in-vivo examinations could not be conducted in this study due to constraints. Thus, future research may focus on assessing the safety and efficacy of these formulations in retinal cell lines and rabbit models. Furthermore, long-term stability studies were not conducted, which can also be considered in future work.

## Competing Interests

 The authors declare that they have no conflict of interest.

## Ethical Approval

 All experiments were authorized by the institutional research ethics committee at Mashhad University of Medical Sciences with IR.MUMS.PHARMACY.REC.1399.004 number.
